# Potential for Hydroclimatically Driven Shifts in Infectious Disease Outbreaks: The Case of Tularemia in High-Latitude Regions

**DOI:** 10.3390/ijerph16193717

**Published:** 2019-10-02

**Authors:** Yan Ma, Arvid Bring, Zahra Kalantari, Georgia Destouni

**Affiliations:** 1 Stockholm University, Department of Physical Geography, 106 91 Stockholm, Sweden; arvid.bring@natgeo.su.se (A.B.); zahra.kalantari@natgeo.su.se (Z.K.); georgia.destouni@natgeo.su.se (G.D.); 2 Bolin Centre for Climate Research, Stockholm University, 114 19 Stockholm, Sweden

**Keywords:** hydroclimatic change, infectious disease, tularemia, critical thresholds, high-latitude regions, Arctic

## Abstract

Hydroclimatic changes may be particularly pronounced in high-latitude regions and can influence infectious diseases, jeopardizing regional human and animal health. In this study, we consider the example of tularemia, one of the most studied diseases in high-latitude regions, which is likely to be impacted by large regional hydroclimatic changes. For this disease case, we use a validated statistical model and develop a method for quantifying possible hydroclimatically driven shifts in outbreak conditions. The results show high sensitivity of tularemia outbreaks to certain combinations of hydroclimatic variable values. These values are within the range of past regional observations and may represent just mildly shifted conditions from current hydroclimatic averages. The methodology developed also facilitates relatively simple identification of possible critical hydroclimatic thresholds, beyond which unacceptable endemic disease levels may be reached. These results call for further research on how projected hydroclimatic changes may affect future outbreaks of tularemia and other infectious diseases in high-latitude and other world regions, with particular focus on critical thresholds to high-risk conditions. More research is also needed on the generality and spatiotemporal transferability of statistical disease models.

## 1. Introduction

Hydroclimatic changes in the landscape, such as in runoff [1], evapotranspiration [2], and often both [3], are being observed and projected regionally and globally, adding to, or occasionally opposing, atmospheric changes in precipitation [4]. Such changes have serious implications for terrestrial water cycling and availability, with particular availability impacts in cold regions, where the water cycle also depends on snow and ice conditions [1]. Hydroclimatic changes can also cause profound and complex shifts in the geographical range, prevalence, and/or severity of some infectious diseases [5,6,7,8,9]. 

The mechanisms of such disease impacts are wide-ranging and may include direct or indirect hydroclimatic influences on abundance of vectors, such as mosquitoes and ticks [10], pathogen survival outside the host [11], host-pathogen interactions that are closely related to community ecology and biodiversity [12,13], dampening of host immunity [14], exposure to water-borne pollution and associated infections [15], including from remobilization of previously frozen pathogens through permafrost thaw under warming [16], and deterioration in health status, e.g., malnutrition and disruption of health systems associated with extreme hydroclimatic events, such as droughts and floods [17]. High-latitude and Arctic regions, where the diversity of animal, plant, and microbial species is low, and where surface temperatures are increasing faster than the global average [18], are subject to particular and in some respects (considering glacier melting and permafrost thaw) more severe and uncertain hydroclimatic changes [19,20,21] linked to health and infectious diseases [16,22,23]. These particularities and disease links make it essential to understand how such changes can affect infectious diseases, and how to identify and quantify future risk shifts due to changing hydroclimate in these high-latitude regions.

Many models have been established to reveal the relations between diseases and hydroclimatic factors. However, the contributions of hydroclimatic changes to predicted disease cases are not directly quantified in these models. In this study, we address this knowledge gap by developing a method for identifying and quantifying hydroclimatic changes and their implications for disease outbreak levels, and possible thresholds to such critical/unacceptable levels, based on a previously established statistical model for the example disease of tularemia in high-latitude areas [24]. Tularemia, caused by the arthropod-borne pathogen *Francisella tularensis*, affects wild animals and humans, and is one of the most researched high-latitude vector-borne diseases likely to be impacted by hydroclimatic change [25]. Its prevalence is verified in Europe, Asia, and America [26] and its natural foci includes high-latitude areas in, e.g., Russia [27], Finland, and Sweden [28]. Climate change is further expected to expand or shift the geographical distribution of tularemia in Russia and the U.S.A. [29,30], and increase the disease burden in high-endemic areas of Sweden [31]. However, contradictory predictions regarding the latter effect have also been published [32]. 

The tularemia model used in this study is based on mosquitoes as the main disease vector in the large boreal forest regions of Alaska, Sweden, Finland, and Russia [33,34,35]. Although it is highly likely that other factors also affect outbreaks, the model has been used successfully to capture a series of annual outbreaks in Dalarna County, Sweden, predicting six out of seven high-incidence years occurring between 1981 and 2007 in this region [24]. These successes indicate that the model has high potential to successfully represent scenarios of possible future tularemia outbreaks under hydroclimatic change in such high-latitude areas, which is a main reason for its selection and being used in the present study. The study also considers and addresses the approach recommended by the World Health Organization (WHO) for assessing climate change impacts on health, by applying the model for possible identification of driver thresholds beyond which unacceptable health hazards may occur under different future hydroclimatic scenarios [36].

## 2. Methods with analytical solution development

The statistical model [24] of annual numbers of humans contracting tularemia is based on identified key variables for mosquito abundance and tularemia-relevant local weather, using available data for the period 1981-2007 in Dalarna County, Sweden. Specifically, the model is as follows:

Tul = EXP(−11 + 0.52 log_2_ Tul_lag_ + 0.54 log_2_ RMA + 0.65 ST_lag_ + 0.012 SP − 0.15 CW) (1)

and it relates the annual number of tularemia cases (Tul) to relative annual mosquito abundance (RMA) (which in turn depends on some periodic hydroclimatic variables of water flow (Q) and temperature conditions (T), as explained further below), summer temperature in the preceding year (ST_lag_), summer precipitation in the same year (SP), winter days with low snow coverage (CW), and number of tularemia cases in the preceding year (Tul_lag_). 

**Figure 1:**
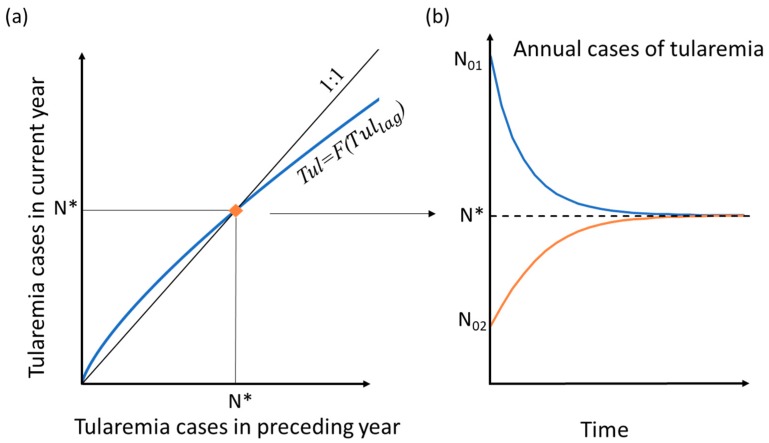
Conceptual diagram of resulting annual number of tularemia outbreaks under some given, unchanging hydroclimatic conditions, as a function of (**a**) corresponding number of outbreaks in the preceding year and (**b**) time. The blue curve in (a) shows the number of tularemia cases in the current year (Tul) as a function of the number of cases in the preceding year (Tul_lag_); the intersection of the blue curve with the black 1:1 line at Tul=Tul_lag_=N* identifies the expected endemic level for the (arbitrary) considered, unchanging hydroclimatic conditions. The blue and orange curves in (b) illustrate the general convergence over time to the endemic level Tul=N* (dashed line) for any initial Tul_lag_ value (N_01_, N_02_) under the given unchanging hydroclimatic setting.

To quantify and readily compare the sensitivity of the number of tularemia cases, Tul, for different hydroclimatic conditions, and ultimately identify possible critical condition thresholds, we derived a model-implicit comparative index in terms of expected endemic levels by rearranging the analytical model Equation (1). This comparative index is the expected number of disease cases that each combination of hydroclimatic conditions will tend to drive the number of tularemia cases towards (asymptotically for temporally stable conditions) based on the underlying model equations (Figure 1). To mathematically derive this index of expected endemic level, the model was first rearranged to represent all the hydroclimate variables by parameter A (Equation (2)), which is a constant in an unchanging hydroclimate setting:

A = G(RMA(Q,T), ST_lag_, SP, CW) (2)

Tul = F(Tul_lag_ ) = A * Tul_lag_^0.75^ (3)

The number of tularemia cases (Tul) in any current/projected year thus becomes a function of a single variable: the number of tularemia cases in the preceding year (Tul_lag_) to the power of 0.75, a constant derived from the rearrangement of Equation (1) through Equation (2).

Equation (3) implies that, for any given (prevailing/projected) combination of hydroclimatic variable values determining the value of A, the value of Tul can be computed from the corresponding value of Tul_lag_ for the preceding year. The associated functional relationship Tul=F(Tul_lag_) is illustrated in Figure 1a. The blue curve (passing through the origin and convex upwards) intersects with the black 1:1 line (of Tul=Tul_lag,_ drawn from the origin with a slope of 1) at some value of Tul=Tul_lag_=N*. The black line represents the theoretical case of unchanging number of tularemia outbreaks between years (Tul=Tul_lag_) and its intersection with the blue curve Tul=F(Tul_lag_) at Tul=Tul_lag_=N* indicates a possible steady state number of outbreaks, N*. Through iteration over succeeding years with any given constant value of A (i.e., constant disease-relevant hydroclimatic conditions) and any initial value of Tul_lag_, either larger (N_01_) or smaller (N_02_) than N*, the resulting number of tularemia outbreaks Tul will in following years converge towards and stabilize at N* (Figure 1b). The resulting value of N* thus represents an expected endemic level for the hydroclimatic conditions considered (determining the value of A). Intersection at the origin, i.e., N*=0, means that the number of annual tularemia outbreaks will tend to vanish over a series of years under the given hydroclimatic conditions. 

Note that the index of expected endemic level N* does not represent the actual tularemia cases in a specific year with weather conditions as in a considered combination of hydroclimatic model variables, since weather does not remain constant over consecutive years. However, the level N* works well as a comparative indicator of how different hydroclimatic factors contribute to disease outbreaks, and how the number of tularemia cases will tend to change driven by changes in long-term average hydroclimate. The latter are much slower than weather changes since, by definition, climate is the long-term average of short-term weather conditions. 

**Figure 2:**
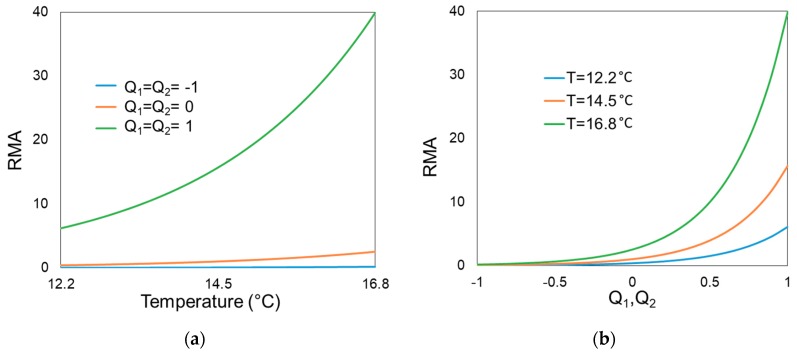
Relative mosquito abundance (RMA) as a function of (**a**) mean temperature (T) 1–7 days before evaluation time t, and (**b**) maximum standardized river flows (in associated standard deviations) Q_1_ and Q_2_ at time periods 36–42 days and 22–28 days, respectively, before time t. The results in (**a**) are for different Q_1_ and Q_2_ values (in associated standard deviations) and the results in (**b**) are for different T values. All Q_1_, Q_2_, and T values considered are within their respective observation-based value ranges.

After developing and using this analytical solution method, we further evaluated the effects of different hydroclimatic variable conditions on disease outbreaks. For this, we calculated the expected endemic level N* for different values of any individual model variable (RMA, ST_lag_, SP, and CW) within its data-given observation range (0.09–39, 12.2–16.8 °C, 143–342 mm, and 0–28 days, respectively) [24], while keeping the other model variables at their median values. For RMA, which is not in itself a hydroclimatic variable but depends on such variables, we also calculated its dependence on the relevant hydroclimatic variable values of maximum standardized river flow for two periods preceding the evaluation time t, denoted Q_1_ and Q_2_ (for 36–42 days and 22–28 days, respectively, before time t), and mean temperature (T) over 1–7 days before time t. This calculation was based on meteorological and hydrological data for Q_1_, Q_2_, and T, obtained from the Swedish Meteorological and Hydrological Institute (www.smhi.se) for the years 1981–2007. Given that any possible extreme daily values of RMA are filtered out when considering the annual median of this variable for use in Equation (1), we calculated the range of annual median RMA for ranges of Q_1_ and Q_2_ between −1 and 1 standard deviation of each variable (Figure 2a). Furthermore, we used the observed summer temperature range (12.3 °C to 16.8 °C) for T (Figure 2b). Since coefficients of Q_1_ and Q_2_ in the model are similar under the same hydroclimate, we only plotted scenarios where Q_1_ is equal to Q_2_. For scenarios where Q_1_ is not equal to Q_2_, derived values of RMA will fall between the two extreme line boundaries (blue and green lines in Figure 2a,b). For these value ranges, the resulting RMA variation is from 0.02 up to 39.9 (Figure 2), which is similar to the previously reported RMA range (0.09–39) [24]. The observation-based ranges of underlying hydroclimatic variables Q_1_, Q_2_, and T considered here thus reproduce a consistent range of annual RMA, as needed for hydroclimatic sensitivity assessment of RMA in the model (Equation (1)).

## 3. Results

**Figure 3:**
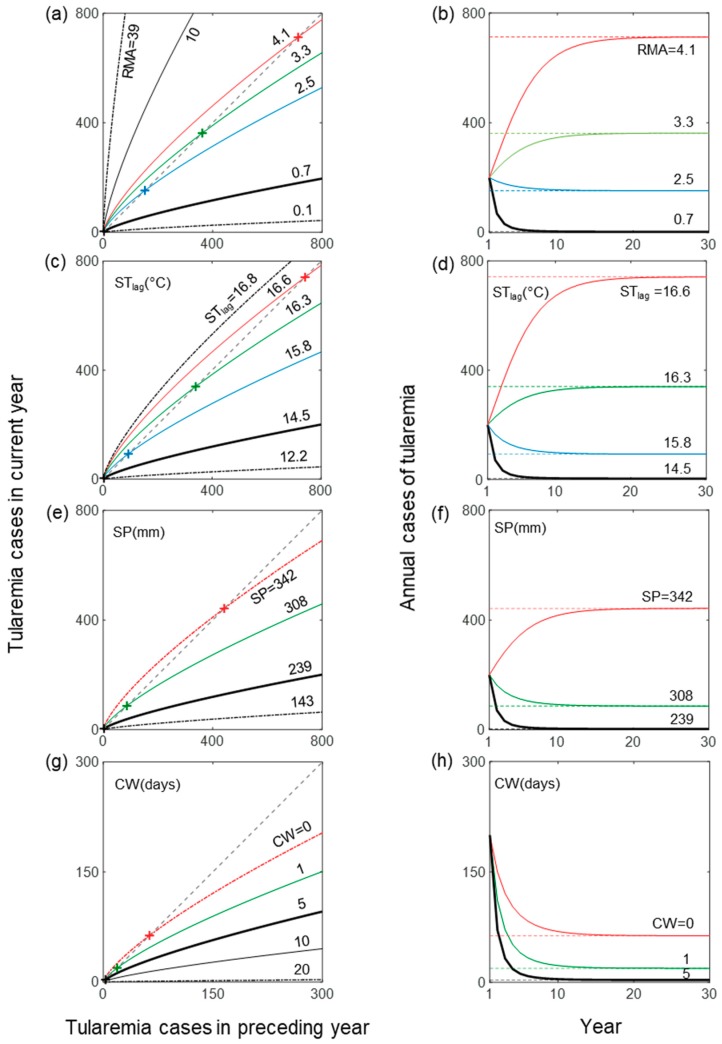
Resulting number of outbreaks and endemic level of tularemia for different hydroclimatic conditions in terms of (**a**,**b**) relative mosquito abundance (RMA) (hydroclimatically determined), (**c**,**d**) summer temperature in preceding year (ST_lag_), (**e**,**f**) summer precipitation (SP), and (**g**,**h**) number of cold winter days with low snow coverage (CW). Panels on the left (right) show tularemia cases (solid colored lines) in current year as a function of cases in the preceding year (time), for different given values of the specific hydroclimate variable considered in each panel, and an initial number of 200 cases (all panels). Dot-dashed lines in the left-hand panels indicate results for observed minimum and maximum hydroclimatic variable values, while the bold line indicates the observed median variable value. The dashed lines in the right-hand panels show the resulting endemic levels (crosses in the left-hand panels).

Figure 3 shows modeled expected endemic levels (crosses in the left-hand panels, dashed lines in the right-hand panels) for different examples of hydroclimatic variable values considered. For all hydroclimatic variable sets, median-range values (indicated by bold black lines in all panels) showed endemic levels close to zero. However, even small variable deviations from these median values can trigger large shifts in endemic levels. Modeled endemic levels are particularly sensitive to the value of RMA, and thereby to the underlying values of Q_1_, Q_2_, and T (Figure 2). For example, a value of 4.1 for RMA, which is higher than the median value, but far from the maximum observed value, resulted in endemic level close to 800 annual cases. This was much higher than the near-zero endemic level for median RMA and also higher than, for example, the resulting endemic level of slightly above 400 for the maximum value of 342 mm for SP. For CW, the highest resulting endemic level was less than 100, which is much lower than the maximum levels that other variables could trigger. 

**Figure 4:**
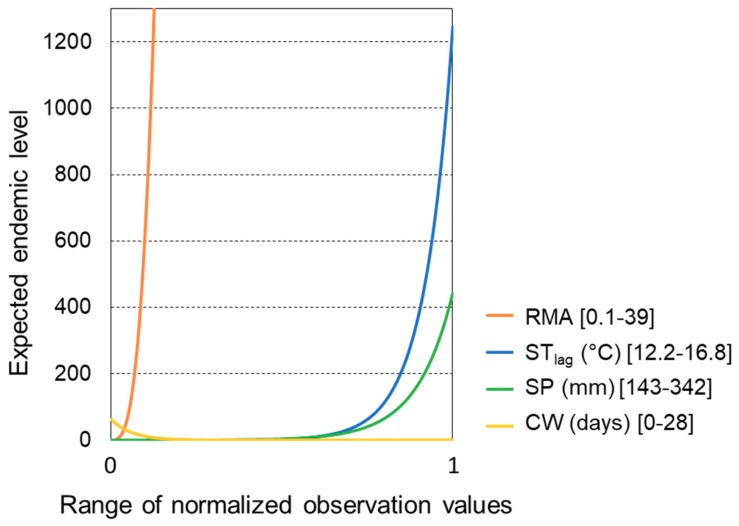
Sensitivity of endemic levels of tularemia to shifts in individual hydroclimate variable values within the range of past observations. The values of all variables are normalized to the same range [0,1], shown on the x-axis. The variables are: relative mosquito abundance (RMA), summer temperature in the preceding year (ST_lag_), summer precipitation in current year (SP), and number of cold winter days with low snow coverage (CW).

To directly compare result sensitivity across all variables, the endemic level response to variable value shifts were plotted over the same standardized value range for all variables (Figure 4). The most dominant variable was then indeed found to be RMA, for which even values far below its maximum observed value yielded very high endemic levels. The next most dominant variables were ST_lag_ and SP, for which variations within the observed range, but close to their respective observed maximum value, raised the endemic level to 1200 and 400 annual cases, respectively. In comparison with the other model variables, CW variation contributed much less to outbreak variation (Figure 4).

**Figure 5:**
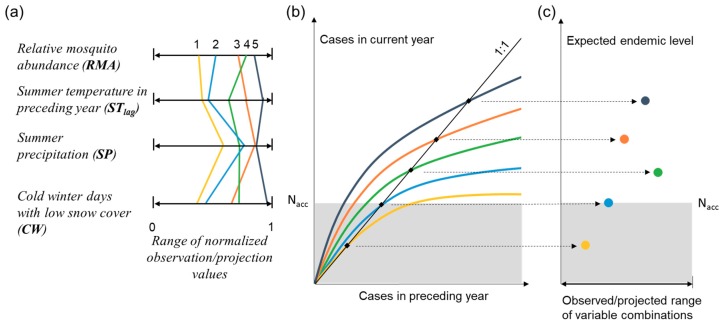
Schematic illustration of how hydroclimatic thresholds to hazardous endemic levels of disease can be identified. The colored curves and symbols show (**a**) examples of hydroclimatic variable value combinations (1–5; different colored lines) within the respective observed/projected value range of each variable; the different points that each line passes through in the four normalized axes represent the values of relevant variables in this hydroclimatic setting; (**b**) number of disease cases as a function of number of cases in the preceding year (as explained in Methods, this function can be obtained by substituting values of the four relevant variables from panel (a) into the model), and endemic level (filled black squares) for the hydroclimatic combination examples (1–5, with similarly colored lines as) in panel (a), compared with the maximum societally acceptable endemic level (N_acc_, upper boundary of gray zone); and (**c**) endemic levels (filled colored circles) for each hydroclimatic combination example (1–5, with similarly colored symbols as the corresponding curves) in panel (a), plotted in relation to the observed/projected variable range (illustrated here for simplicity in just one dimension, the x-axis, whereas the actual range space is multi-dimensional depending on the number of hydroclimatic variables considered).

Endemic level can also be used as an index for identification of hydroclimatic thresholds to critical/unacceptable health hazards (Figure 5). As a basis for such identification, a highest societally acceptable endemic level (N_acc_) needs to be defined (upper boundary of gray area in Figure 5b,c). Combinations of observed or projected hydroclimate variable values leading to endemic levels at or around N_acc_ can then be identified as critical thresholds (e.g., variable combination number 2, light blue in Figure 5) between hydroclimatic conditions that are acceptable (e.g., yellow combination number 1) and those that are high-risk (green, orange, dark blue combinations 3–5) from a health/disease perspective. Acceptable endemic level here is different from the alert thresholds for epidemic-prone conditions of other diseases defined by WHO for EWARN (Early Warning Alert and Response Network) [37], because it is not the actual cases that will happen in a certain year but a comparative indicator of the tendency of tularemia cases driven by different hydroclimatic conditions. Therefore, the acceptable level of this indicator has to be further studied and defined by relevant agencies, based on estimated health risks associated with disease outbreak numbers approaching or becoming greater than this acceptable endemic level.

## 4. Discussion

Using a previously tested and validated statistical disease model, we computed model-implicit expected endemic levels of tularemia with hydroclimatic variable values within their respective ranges of observations. We demonstrated that the computed expected endemic level can be used as a comparative index for analyzing the sensitivity of tularemia outbreaks to hydroclimatic conditions and for identifying critical hydroclimatic thresholds to high-risk outbreak conditions. Our analysis revealed that tularemia outbreaks are most sensitive to relative mosquito abundance (RMA) and associated underlying periodic hydroclimatic conditions of flow (Q_1_ and Q_2_) and mean temperature (T), followed by summer temperature in the preceding year (ST_lag_) and summer precipitation in the current year (SP). Number of cold winter days with low snow coverage (CW) has the least influence on outbreaks and is the only hydroclimatic variable with a negative relationship with outbreaks, i.e., number of outbreaks and endemic level decreased with increasing CW (Figure 3g,h, Figure 4 ).

Although only 370 people were diagnosed with tularemia in the study area of Dalarna County over the 27-year study period (1981–2007) used for development of the statistical model [24], our analysis revealed that certain combinations of relevant hydroclimatic variables, with values within the historically observed ranges, have high potential to trigger many more outbreaks. Projections of future climate suggest that the range of hydroclimatic variations may move outside the historical range for many locations [38], implying even more threatening future conditions.

Previous studies have also investigated methods for determining the sensitivity of infectious diseases to hydroclimate change. Time-series studies, especially correlation or regression analyses [39,40], and geographical comparisons [41], have been used for this purpose. A recent study [41] reports advantages of geographical comparisons over time-series analysis, such as the possibility of using short data time series. However, that study used relative sensitivity as a statistical indicator of climate-sensitive infectious diseases, revealing the impact of only a single variable without considering temporal autocorrelation effects, whereby high incidence in one month or year might increase transmission potential in subsequent months or years. In contrast, the use of endemic level as an indicator, based on a validated statistical disease model, considers both interactions among several variables and temporal autocorrelations. Moreover, endemic level may be an effective comparative indicator across different regions and/or different infectious diseases, if validated statistical models are available for identifying endemic levels in comparable ways. 

Here we only considered historic variable observation ranges, but the method for identifying endemic level and using it as an index for further identification of threshold hydroclimatic conditions can also be used with projected possible future values of the relevant hydroclimatic variables. Endemic levels and probability of exceeding hydroclimatic thresholds can then be compared between past conditions and projected future scenarios. Such comparisons will be useful for studying whether, how, and to what extent future hydroclimate changes may affect disease outbreaks and associated risks. 

The WHO has performed a quantitative risk assessment of the effects of climate change on mortality caused by diarrhea, malaria, and dengue fever, using outcome-specific models to estimate future infectious disease-induced mortality with and without climate change [42]. The differences between the two scenarios are then considered to be climate-driven impacts. As in the present study, the aim of the WHO assessment is to predict health impacts of climate change, but its approach requires large amounts of data and introduces uncertainty through choices of baseline climate and socioeconomic development, education, and technology scenarios for the future. In comparison, the method developed here only requires data on relevant hydroclimatic conditions for indicating likely trends in tularemia cases under different future situations. 

Limitations of the method presented here include various constraints in the underlying statistical model, such as not representing changes in human behavior. For example, incorrect prediction by the model of tularemia occurrence in year 1987 may have been caused by reduction in forest berry picking and hunting due to the Chernobyl disaster in the preceding year, leading to minimized exposure to tularemia [24]. Moreover, the method assumes independence of the considered hydroclimatic variables from societal factors that, in reality, may affect both hydroclimate and the tularemia outbreaks, such as climate mitigation-adaptation measures. The time that it may take for the number of outbreaks to approach expected endemic levels, driven by long-term hydroclimatic changes, is also not considered, and may vary for different hydroclimatic conditions. This modeling limitation can be overcome by considering some convergence rate, although validation of such assumed rates will be difficult under long-term hydroclimatic variability and change.

Furthermore, regional statistics-based disease models, such as that used here for tularemia, may not be directly transferable to other geographical regions and/or scales. One way forward is then to use specifically calibrated models for different regions/scales. For example, region-specific models for seven high-risk parts of Sweden have been established and show high capability in predicting regional outbreaks from 1984 to 2012, with potential of using such region/scale-specific models to analyze hydroclimatically driven shifts from the past to the future in each region and compare model coefficients and results among the regions [43]. 

The purpose of the present work was to develop a method for interpreting the effect of hydroclimatic factors from statistical models, by which hydroclimatic data can provide a straightforward picture of comparative risk levels. The method has the flexibility to be applied to different statistical disease models, but the extent to which the risk can be analyzed relies on the availability of such validated models. The model and validation limitations so far call for further research on how hydroclimate changes affect infectious disease outbreaks across regions and scales, with particular attention to potential critical thresholds and to the generality and spatiotemporal transferability of statistical disease models.

## 5. Conclusions

We developed and tested a method that analyzes the sensitivity of infectious diseases to hydroclimatic changes and can assist in identifying critical hydroclimatic thresholds to unacceptable health hazards. A statistical model of tularemia was used as an example. A key contribution of our approach is in identifying comparative endemic levels to which disease outbreak numbers will tend to converge under different hydroclimate conditions. The case results showed high sensitivity of tularemia outbreaks to certain threshold combinations of hydroclimatic variable values within the range of past regional observations, although average conditions may shift under ongoing climate change, increasing the risk. Critical hydroclimatic thresholds can be identified, beyond which the associated expected endemic levels are not societally acceptable. Further research is needed on how such acceptable levels can be defined and whether projected climate change tends to drive hydroclimatic conditions towards and beyond such critical thresholds. The generality and spatiotemporal transferability of statistical disease models, such as that for tularemia used in this study, also need to be investigated in further research.
